# Peripheral non-enzymatic antioxidants in patients with schizophrenia: a case-control study

**DOI:** 10.1186/s12888-020-02635-8

**Published:** 2020-05-15

**Authors:** Zhe Lu, Tianyang Wen, Yingtan Wang, Weijing Kan, Guanglei Xun

**Affiliations:** 1 Cheeloo College of Medicine, Shandong University, 44# Wenhua Western Road, Jinan, 250012 China; 2 Shandong Mental Health Center, 49# Wenhua Eastern Road, Jinan, 250014 China; 3 Department of Mental Health, Jining Medical University, 133# Hehua Road, Beihu New District, Jining, 272067 China; 4 Beijing An Ding Hospital, Capital Medical University, China National Clinical Research Center for Mental Disorders, Beijing, People’s Republic of China

**Keywords:** Schizophrenia, Uric acid, Albumin, Total bilirubin

## Abstract

**Background:**

Recent studies show that oxidative stress is associated with the pathogenesis of schizophrenia. There are two major types of antioxidant systems in vivo, namely enzymatic antioxidants and non-enzymatic antioxidants. This study investigated differences of non-enzymatic antioxidants between schizophrenia patients and healthy controls.

**Methods:**

Peripheral UA, ALB, and TBIL of 107 schizophrenic patients in the acute stage and 101 in the remission stage were measured respectively, so were 273 healthy controls.

**Results:**

The levels of UA (*P* = 0.020) and TBIL (*P* < 0.001) of schizophrenic patients in the acute stage were higher than those of healthy controls, while the level of ALB (*P* < 0.001) was lower. Similar results were detected form schizophrenic patients in the remission stage. Schizophrenic patients in the acute stage were divided into antipsychotics-use subgroup (*n* = 56) and antipsychotics-naïve/free subgroup (*n* = 51). The level of UA (*P* = 0.001) in the antipsychotics-use subgroup was higher than that in the antipsychotics-naïve/free subgroup, while the level of TBIL (*P* = 0.002) was lower than that in the antipsychotics-naïve/free subgroup. Seventy-seven schizophrenic patients in the acute stage were followed up, and there was no significant difference in the level of UA before and after treatment, but levels of ALB (*P* < 0.001) and TBIL (*P* < 0.001) decreased significantly after the treatment.

**Conclusion:**

This study demonstrated that the dysfunction of the peripheral non-enzymatic anti-oxidation system might be involved in the pathogenesis of schizophrenia.

## Background

Up to now, the pathogenesis of schizophrenia (SCZ) remains unclear. It has been proved that biological, psychological, and social factors contribute to the pathogenesis of SCZ. Moreover, previous studies suggested that oxidative stress was related to the pathogenesis of SCZ [1].

Oxidative stress was defined as the imbalance between reactive oxygen/nitrogen species (ROS/RNS) and antioxidant protection systems. Increasing oxidative stress leads to the deleterious oxidation and chemical modification of biomacromolecules such as lipids, DNA, and proteins [2, 3]. Antioxidant refers to any compound which can lower oxidative stress by depleting molecular oxygen or decreasing its local concentration, removing pro-oxidative metal ions, trapping aggressive reactive oxygen species, scavenging chain-initiating radicals, breaking the chain of a radical sequence or quenching singlet oxygen [4, 5]. Redox homeostasis of the cell is ensured by a complex endogenous antioxidant defense system, which includes enzymatic antioxidants and non-enzymatic antioxidants [6, 7]. The former is the main antioxidant system in the cell with three key enzymes: superoxide dismutase, catalase, and glutathione peroxidase; the latter is the main antioxidant system in the extracellular fluid (such as plasma, cerebrospinal fluid), mainly including vitamin A and C, tocopherol, glutathione, uric acid (UA), albumin (ALB), bilirubin [4, 8]. Nervous tissue is extremely sensitive to oxidative damage caused by ROS or RNS. To be specific, the mitochondrial aerobic respiration, cellular structural component peroxidation, and phagocytosis of microglia can produce a large amount of ROS and RNS to cause oxidative damage to brain tissue [9]. The mechanism of oxidative stress in SCZ is yet not clear, but increasing pieces of evidence suggest that oxidative stress involves in the pathophysiology of SCZ [10–13].

Recent studies show an elevated level of oxidative stress indicators in SCZ. An autopsy study conducted by Yao et al. [14] found that level of nitric oxide in the caudate nucleus of schizophrenic patients was significantly higher than that of healthy controls, indicating that there was a difference in oxidative stress in different brain regions of schizophrenic patients. Radonjic et al. ‘s result in the animal study further confirmed this viewpoint [15]. They fed perinatal rats with phencyclidine to establish a model of SCZ and found an evident increase in lipid peroxidation level in the hippocampus and thalamus. In summary, patients with SCZ are usually in a state of high oxidative stress. Specifically, their oxidative stress indicators are significantly higher than those of healthy people, and the oxidation status of different brain regions varies.

Various factors may influence antioxidant status. First of all, the ageing process is linked with oxidative injury [16]. Next, diet, caloric intake, and alcohol can affect both the antioxidant system and the production of free radicals [17]. Besides, the cigarette may contribute directly to oxidative stress and decreases in antioxidants because of containing many pro-oxidants [18]. On the other hand, nicotine, as an ingredient in cigarette smoke, has been shown antioxidant properties in several vivo studies [19]. Concerning antipsychotics, there is no common understanding towards that to what extent the antipsychotics affect oxidative stress. Al-Chalabi et al. [20] found that olanzapine could improve plasma total antioxidant status and alleviate lipid peroxidative injury. Nevertheless, Eftekhari et al. [21] found in animal experiments that olanzapine could induce oxidative stress and hepatotoxicity, which was associated with the CYP450 enzyme. A recent study pointed out that olanzapine and clozapine had a higher antioxidant ability than risperidone, quetiapine, ziprasidone, and haloperidol [22]. The effects of typical and atypical antipsychotics on oxidative stress are different. The former may aggravate oxidative damage, while the latter may improve oxidative state [5, 22–24]. Nevertheless, whether the effects are direct or indirect requires further study. All in all, these inconsistencies indicate that oxidative stress in SCZ may be independent of antipsychotic treatment.

Current studies on oxidative stress in SCZ mainly focus on enzymatic antioxidants, while limited studies have been carried out on non-enzymatic antioxidants. Some studies discovered that plasma non-enzymatic antioxidants (uric acid, bilirubin, and albumin) in SCZ are lower than those of healthy controls. Reddy et al. found that levels of UA, total bilirubin (TBIL), and ALB in SCZ were significantly lower than those of healthy controls and were affected by gender [25]. Widschwendter et al. carried out a retrospective study in 2016, and found that level of plasma TBIL in schizophrenic patients was significantly lower than the baseline at the end of the 2nd and the 4th week after treatment, while the latter decreased more substantially, which was correlated with positive subscale score of Positive and Negative Syndrome Scale (PANSS) [26]. However, these findings were not reproduced in another similar study [10].

Previous studies on non-enzymatic antioxidants of patients with SCZ are limited and conflicting. Therefore, the objective of the present study is to investigate whether there are any differences in peripheral levels of non-enzymatic antioxidants between patients with SCZ and healthy individuals, as well as to observe the effect of antipsychotics on levels of non-enzymatic antioxidants.

## Methods

### Study population

A total of 107 schizophrenic patients (40 males, 67 females; mean age: 34.03 ± 11.03 years, range: 18–57 years) in the acute stage and 101 schizophrenic patients (37 males, 64 females; mean age: 35.36 ± 11.31 years, range: 18–57 years) in remission stage were screened from outpatients and inpatients in Shandong Mental Health Center during May 2018 to May 2019. The control group consisted of 273 healthy individuals (93 males, 180 females, mean age: 34.92 ± 9.22 years, range: 23–60 years) were invited with matched ages and genders. One hundred seven schizophrenic patients in the acute stage were followed up for 12 weeks, and their levels of non-enzymatic antioxidants were measured again at the end. Inclusion Criteria and exclusion criteria were as follows:

Inclusion criteria for schizophrenic patients in the acute stage (SCZ-AS): (1) 18–60 years of age, Han Chinese; (2) Diagnosed with SCZ based on Diagnostic and Statistical Manual of Mental Disorders, fifth edition (DSM-5) criteria; (3) PANSS total score ≥ 70.

Inclusion criteria for schizophrenic patients in the remission stage (SCZ-RS): (1) 18–60 years of age, Han Chinese; (2) Previously diagnosed with SCZ based on.

DSM-5; (3) PANSS total score ≤ 60, score ≤ 4 on 7 PANSS items (delusions, conceptual disorganization, hallucinatory behavior, suspiciousness/persecution, hostility, uncooperativeness, and poor impulse control) and Clinical Global Impressions-Severity of Illness (CGI-S) score ≤ 4 at least 6 months [27].

Inclusion criteria for healthy controls (HC): (1) 18–60 years of age, Han Chinese; (2) No family history of psychiatric disorders.

Exclusion criteria applied for all groups: (1) Combined with organic brain diseases or brain trauma. (2) Hypertension, diabetes, gout or liver, kidney, biliary, and other physical diseases or abnormal renal and liver function. (3) Combined with other mental disorders. (4) Positive in urine pregnancy test or lactating females. (5) Modified electroconvulsive therapy (MECT) treatment within 4 weeks, or long-acting antipsychotics treatment within 6 months; (6) Taking antioxidants or neurotrophic drugs within 12 weeks before and during enrollment; (7) Alcohol and other substance usages.

The study protocol was approved by the Clinical Research Ethics Committee of Shandong Mental Health Center and is compliant with the Code of Ethics of the World Medical Association (Declaration of Helsinki). Informed written consent was obtained from all participants or their legal guardians after a complete and extensive description.

### Measurements

The clinical psychiatric symptoms in schizophrenic patients were assessed by the Chinese PANSS. The psychiatrists (all authors) were simultaneously trained in the use of the PANSS before this study was initiated. After training, repeated assessments indicated that the inter-observer correlation coefficient was maintained at greater than 0.80 for the PANSS total score.

Five milliliters of fasting venous blood samples were drawn from participants between 7:00 am and 7:30 am. Serum samples were separated by centrifugation (3000 rpm for 10 min at 20 °C) and stored at − 80 °C. Peripheral levels of UA, ALB, and TBIL were detected by Roche Cobas C702 automatic biochemical analyzer (Swiss Roche Diagnostics Co., Ltd.) according to the manufacturer’s instructions.

### Statistical analysis

The results were expressed as mean ± standard deviation. Differences in continuous variables among groups were assessed by the independent samples t-test and one-way analysis of variance. The chi-square test was applied to categorical data such as gender. The paired samples test was adopted to evaluate changes of UA, ALB, TBIL, and PANSS total scores before and after treatment. The *Pearson* correlation analysis was adopted to analyze the relationship between antioxidant levels and PANSS. *P*-values < 0.05 were considered statistically significant.

## Results

### Demographic and clinical data

The demographic and clinical characteristics of participants are summarized in Table 1. There were no differences in demographic data (gender and age) among the three groups. The differences in clinical data (smoking history, family history, and duration of illness) between the SCZ-AS group and SCZ-RS group were not significant.

**Table 1:** Demographic characteristics and clinical data (Mean ± SD) of patients with schizophrenia and healthy controls

Variables	SCZ-AS(*n* = 107)	SCZ-RS (*n* = 101)	HC (*n* = 273)	*F/t/χ*^*2*^	*P*
Gender (male/female)	40/67	37/64	93/180	1.386	0.500
Age (years)	34.03 ± 11.03	35.36 ± 11.31	34.92 ± 9.22	0.467	0.627
Smokers/non-smokers	10/97	11/90	26/247	0.185	0.912
Family history (positive/negative)	25/82	22/79	–	0.074	0.785
Duration of illness (months)	96.70 ± 80.48	108.23 ± 96.605	–	0.995	0.321
Drug naïve or free/ Drug use	51/56	–	–	–	–
PANSS total scores	93.21 ± 16.46	45.44 ± 8.40	–	25.531	<0.001

*SCZ-AS* schizophrenic patients in the acute stage, *SCZ-RS* schizophrenic patients in the remission stage, *HC* healthy control, *PANSS* Positive and Negative Syndrome Scale

### SCZ-AS vs. HC

Compared with the HC group, the levels of UA (*t* = 3.170, *P* = 0.020) and TBIL(*t* = 8.166, *P*<0.001) in the SCZ-AS group were higher, while the level of ALB(*t* = − 13.188, *P*<0.001) was lower. There was no significant difference in the level of UA between males in the HC and SCZ-AS group. Males’ level of ALB (*t* = − 6.435, *P*<0.001) in the SCZ-AS group was lower than that of the HC group, while the level of TBIL (*t* = 4.517, *P*<0.001) was higher. The levels of UA (*t* = 2.937, *P =* 0.004) and TBIL (*t* = 7.984, *P*<0.001) of females in the SCZ-AS group were higher than those of the HC group, and level of ALB (*t* = − 11.841, *P*<0.001) was lower.

SCZ-AS were divided into antipsychotics-use (AU) subgroup and antipsychotics-naïve/free (ANF) subgroup (unmedicated first-episode schizophrenia or no antipsychotics was used within 8 weeks) and then compared with the HC group. It was observed that levels of UA in the ANF subgroup (*P* = 0.001) and HC group (*P*<0.001) were lower than those of the AU subgroup, and there was no significant difference in the level of UA between the ANF subgroup and the HC group. Levels of ALB of AU subgroup (*P*<0.001) and ANF subgroup (*P*<0.001) were lower than those of the HC group, and there was no significant difference in the level of ALB between the ANF subgroup and AU subgroup. Levels of TBIL of AU (*P*<0.001) subgroup and ANF subgroup (*P*<0.001) were higher than those of the HC group, and the level of TBIL in the ANF subgroup was higher than that of AU subgroup (*P* = 0.002). Analogous results were obtained when analyzing the male and the female separately (Table 2).

**Table 2:** Peripheral levels of uric acid, albumin, and total bilirubin in schizophrenic patients in the acute stage and healthy controls

Group	UA (μmol/L)	ALB (g/L)	TBIL (μmol/L)
SCZ-AS(n = 107)	308.29 ± 78.71^a^*	42.79 ± 3.55^a^***	18.43 ± 9.00^a^***
Male(*n* = 40)	350.45 ± 66.54	44.02 ± 3.36^a^***	19.74 ± 10.89 ^a^***
Female(*n* = 67)	284.72 ± 75.45 ^a^**	42.05 ± 3.48 ^a^***	17.65 ± 7.65 ^a^***
AU(n = 56)	330.32 ± 81.69 ^a^***^,b^**	43.13 ± 3.17^a^***	16.56 ± 5.37^a^***^,b^**
ANF(n = 51)	286.19 ± 68.94	42.40 ± 3.92 ^a^***	20.47 ± 11.49 ^a^***
HC(n = 273)	283.41 ± 68.61	48.01 ± 3.28	10.87 ± 5.18
Male(*n* = 93)	338.69 ± 52.42	48.93 ± 4.29	12.97 ± 6.24
Female(*n* = 180)	254.84 ± 57.72	47.54 ± 2.49	9.78 ± 4.16

*P < 0.05; **P < 0.01; ***P < 0.001

^a^ Compared with the healthy controls group. ^b^ Compared with the antipsychotics-naïve/free subgroup. *SCZ-AS* schizophrenic patients in the acute stage, *AU* antipsychotics-use, *ANF* antipsychotics-naïve/free, *HC* healthy control, *UA* uric acid, *ALB* albumin, *TBIL* total bilirubin

### SCZ-RS vs. HC

Compared with the HC group, the levels of UA (*t* = 4.125, *P*<0.001) and TBIL (*t* = 5.258, *P*<0.001) in the SCZ-RS group were higher, while the level of ALB (*t* = − 21.616, *P*<0.001) was lower. Males’ level of ALB (*t* = − 10.213, *P*<0.001) in the SCZ-RS group was lower than that of the HC group, while levels of TBIL (*t* = 2.613, *P* = 0.010) and UA (*t* = 3.439, *P* = 0.001) was higher. The levels of UA (*t* = 2.937, *P* = 0.002) and TBIL (*t* = 7.984, *P*<0.001) of females in the SCZ-RS group were higher than those of the HC group, and level of ALB (*t* = − 11.841, *P*<0.001) was lower (Table 3).

**Table 3:** Peripheral levels of uric acid, albumin, and total bilirubin in schizophrenic patients in the remission stage and healthy controls

Group	UA (μmol/L)	ALB (g/L)	TBIL (μmol/L)
SCZ-RS(n = 101)	323.19 ± 87.48***	40.29 ± 2.41***	14.39 ± 7.03***
Male(*n* = 37)	384.24 ± 73.49 **	41.41 ± 1.98 ***	16.72 ± 9.69 **
Female(*n* = 64)	287.89 ± 74.87 **	39.64 ± 2.41 ***	13.03 ± 4.45 ***
HC(n = 273)	283.41 ± 68.61	48.01 ± 3.28	10.87 ± 5.18
Male(n = 93)	338.69 ± 52.42	48.93 ± 4.29	12.97 ± 6.24
Female(n = 180)	254.84 ± 57.72	47.54 ± 2.49	9.78 ± 4.16

*P < 0.05; **P < 0.01; ***P < 0.001

*SCZ-RS* schizophrenic patients in remission stage, *HC* healthy control, *UA* uric acid, *ALB* albumin, *TBIL* total bilirubin

### Comparison in levels of UA, ALB, TBIL, and PANSS between pre- and post-treatment in SCZ-AS group

A continuous real-world observation (12 weeks) of before and after treatment were performed on SCZ-AS. In this study, none limitation was conducted on the treatment (detail medication data was showed in Table 4), so that 30 patients in SCZ-AS group were dropped out from this part because of various reasons (such as using hypotensor, lipid-lowing drugs, hypoglycemic agents or antioxidants like vitamin E, receiving MECT, with abnormal liver function and so on).

**Table 4:** Medication data

	Remission group		AU group		Pre- and post-treatment	
	cases	doses (mg/day)	cases	doses (mg/day)	cases	doses (mg/day)
Antipsychotics						
Perphenazine	3	17.33 ± 8.33	5	17.60 ± 11.52	6	19.33 ± 10.86
Sulpiride	–	–	1	600.00	–	–
Risperidone	45	5.36 ± 1.32	26	4.52 ± 1.89	53	4.86 ± 1.55
Amisulpride	7	885.71 ± 302.37	9	600.00 ± 300.00	14	650 ± 295.48
Olanzapine	10	13.50 ± 2.42	16	14.38 ± 8.39	18	14.44 ± 4.50
Ziprasidone	2	140 ± 28.28	4	80 ± 46.19	2	100 ± 84.85
Quetiapine	7	285.71 ± 89.97	9	327.78 ± 207.83	14	296.43 ± 171.49
Clozapine	13	153.85 ± 96.74	12	176.04 ± 162.23	10	111.25 ± 110.62
Aripiprazole	16	15.31 ± 9.74	22	17.05 ± 7.82	22	18.09 ± 10.08
Mood stabilizers						
Valproate	3	700.00 ± 255.84	2	700.00 ± 278.65	5	675.56 ± 245.09
Lithium	2	425.00 ± 206.16	1	560.00 ± 251.00	3	478.57 ± 191.17
Lamotrigine	–	–	1	37.50	–	–
Antidepressants						
Escitalopram	3	10.00 ± 5.00	–	–	4	10 ± 4.08
Fluvoxamine	–	–	1	75	–	–
Sertraline	2	50.00	5	60.00 ± 22.36	3	50.00
Paroxetine	2	40.00	–	–	1	20.00
Trazodone	1	50.00	–	–	–	–

*AU* antipsychotics-use

There was no significant difference in the level of UA before and after treatment, and both levels of ALB and TBIL decreased after treatment. The AU subgroup reached comparable results, but there was no significant difference in the level of TBIL in males in the AU subgroup before and after treatment. The level of UA increased after treatment in the ANF subgroup, while both levels of ALB and TBIL decreased. There were no significant differences in levels of UA, ALB, and TBIL in males in the ANF subgroup before and after treatment. The difference of UA level in females in the ANF subgroup before and after treatment was close to significance, and both levels of ALB and TBIL decreased after treatment (Table 5).

**Table 5:** Peripheral levels of uric acid, albumin, total bilirubin, and PANSS total scores in schizophrenia before and after treatment

Group	Before				After				*t*_*1*_*/t*_*2*_*/t*_*3*_*/t*_*4*_	*P*_*1*_*/P*_*2*_*/P*_*3*_*/P*_*4*_
	UA (μmol/L)	ALB (g/L)	TBIL (μmol/L)	PANSS	UA (μmol/L)	ALB (g/L)	TBIL (μmol/L)	PANSS		
SCZ (*n* = 77)	305.47 ± 78.18	42.61 ± 3.67	17.92 ± 7.32	93.91 ± 17.53	318.00 ± 85.24	39.99 ± 2.23	13.33 ± 5.14	45.19 ± 8.57	1.551/23.300/6.405/5.696	0.125/<0.001/<0.001/<0.001
Male (n = 27)	348.81 ± 64.89	43.65 ± 3.34	17.75 ± 5.49	90.00 ± 18.82	374.00 ± 77.57	41.03 ± 1.77	14.66 ± 6.02	46.07 ± 9.68	1.791/11.931/3.740/2.266	0.085/0.001/0.032/<0.001
Female (*n* = 50)	282.06 ± 75.17	42.04 ± 3.74	18.01 ± 8.20	96.02 ± 16.61	287.76 ± 73.59	39.43 ± 2.26	12.61 ± 4.50	44.72 ± 7.97	0.580/5.148/5.456/20.657	0.564/<0.001/<0.001/<0.001
AU (*n* = 36)	328.69 ± 78.78	43.29 ± 3.31	16.40 ± 5.74	99.03 ± 20.88	329.53 ± 95.18	40.15 ± 2.16	12.84 ± 3.84	46.28 ± 8.42	0.063/5.807/3.985/14.668	0.950/<0.001/<0.001/<0.001
Male (*n* = 15)	359.60 ± 63.40	44.25 ± 2.89	16.99 ± 3.88	93.27 ± 23.26	381.93 ± 83.73	40.79 ± 1.23	14.35 ± 4.04	46.87 ± 8.55	1.220/4.618/1.728/8.126	0.242/<0.001/0.106/<0.001
Female (*n* = 21)	306.62 ± 82.58	42.60 ± 3.48	15.98 ± 6.83	103.14 ± 18.49	292.10 ± 86.11	39.69 ± 2.56	11.76 ± 3.37	45.86 ± 8.51	0.801/3.791/3.888/12.802	0.433/0.001/0.001/<0.001
ANF (*n* = 41)	285.07 ± 72.60	42.00 ± 3.89	19.25 ± 8.32	89.41 ± 12.58	307.88 ± 75.19	39.86 ± 2.30	12.84 ± 6.08	44.24 ± 8.68	2.366/3.592/4.262/20.221	0.023/0.001/<0.001/<0.001
Male (*n* = 12)	335.33 ± 66.93	42.90 ± 3.83	19.25 ± 8.32	85.92 ± 10.79	364.08 ± 71.46	41.33 ± 2.31	12.84 ± 6.08	45.08 ± 11.24	1.264/1.269/1.476/9.456	0.232/0.231/0.168/<0.001
Female (*n* = 29)	264.28 ± 65.10	41.63 ± 3.92	19.47 ± 8.88	90.86 ± 13.15	284.62 ± 64.48	39.25 ± 2.04	13.23 ± 5.14	43.90 ± 7.59	2.007/3.500/4.136/18.207	0.055/0.002/<0.001/<0.001

*AU* antipsychotics-use, *ANF* antipsychotics-naïve/free, *UA* uric acid, *ALB* albumin, *TBIL* total bilirubin, *PANSS* Positive and Negative Syndrome Scale

t1/t2/t3/t4 and P1/P2/P3/P4 are respectively the statistic of UA, ALB, TBIL, and PANSS

There were significant differences in PANSS total scores between pre- and post-treatment. Furthermore, the relationship between PANSS total scores and antioxidant levels was not significant whether in acute stage or remission stage (acute stage: UA, *r* = − 0.003, *P* = 0.975; ALB, *r* = − 0.042, *P* = 0.666; TBIL, *r* = − 0.033, *P* = 0.737; remission stage: UA, *r* = 0.149, *P* = 0.136; ALB, *r* = 0.039, *P* = 0.695; TBIL, *r* = − 0.196, *P* = 0.050) (Table 5).

## Discussion

SCZ has a complex pathophysiological mechanism associated with free radical-mediated neurotoxicity [8]. The effectiveness of the antioxidant defense system on ROS depends not only on its enzymatic component but also on the non-enzymatic composition [28]. Non-enzymatic antioxidants are mainly composed of albumin, bilirubin, and uric acid, which can alleviate oxidative stress by chelating with metal ions and directly capturing radicals in hydroxyl and carbon center, accounting for more than 85% of total plasma antioxidant capacity. Clinically, those indicators can be monitored to detect the presence of oxidative stress damage in vivo [29, 30].

UA, the end product of purine metabolism, proved its ability to scavenge reactive radicals and protect the erythrocyte membrane from lipid peroxidation via reacting with peroxynitrite intermediates, nitric oxide, and peroxyl radical to inactivate them [31]. Appropriately increased UA can enhance the body’s antioxidant capacity. In this study, the level of UA in SCZ patients was higher than that of HC both in the acute and remission stage. Then the SCZ-AS group was reclassified into the AU subgroup and ANF subgroup. The results showed that the level of UA in the AU subgroup was higher than that of the ANF subgroup and HC group, and there was no difference in the level of UA between the HC group and ANF subgroup. It suggests that the elevated level of UA may be related to the use of antipsychotics. This study also noticed that there was no statistical difference in the level of UA before and after treatment in the AU subgroup, but the level of UA increased after treatment in the ANF subgroup, which further confirmed the conclusion that the use of antipsychotics could increase the level of UA. The result was inconsistent with the studies of Reddy et al. and Yao et al. [25, 32]; the cause might be the effects of antipsychotics were not excluded in their studies.

ALB is an endogenous antioxidant with radical scavenging properties that inhibits lipid peroxidation by binding metal ions responsible for the generation of most of the reactive oxygenated radical species [4]. In this study, whether in acute or remission stage, level of ALB in SCZ was lower than that of HC, and decreased after treatment, which was consistent with the previous study [33], indicating that ALB in patients with SCZ was constantly persistently consumed in acute and remission stage. Even though albumin levels decreased in the acute and remission stage as well as after treatment, the decline was modest, and the albumin levels of patients with SCZ in every stage were still in the normal range. It may be due to the liver generating albumin continuously. Results of a five-year follow-up study of antioxidants levels in SCZ conducted by Dag K. Solberg et al. showed that differences in ALB levels between schizophrenic patients in the remission stage and healthy individuals were not significant [34], which was not in coincidence with our result. It may be due to the observation duration of this study was relatively short that the patients might be still in an acute oxidative stress stage. Besides, there was no significant difference in the level of ALB between the AU subgroup and ANF subgroup, suggesting that antipsychotics had little effect on the level of ALB.

Bilirubin is the end product of heme-catabolism that participates in the antioxidative mechanisms by efficiently scavenging peroxyl radicals and acting as a chain-breaking antioxidant [35]. In this study, it was noted that the level of TBIL in SCZ was higher than that of HC, both in the acute and remission stage, which was confirmed by another recent study [34]. The elevated serum bilirubin may be a result of the increasing fragility of the erythrocyte membrane under oxidative stress [33, 36]. At the same time, the pro-oxidant effect of heme-oxygenase may outrun the antioxidant property of bilirubin [37]. Contrary to our results, previous studies derived that level of TBIL in SCZ was lower than that of HC [25, 32, 38–40], which might be caused by the heterogeneity of the sample. Again, this study points out that level of TBIL in the AU subgroup was lower than that of the ANF subgroup and decreased after treatment. It indicated that antipsychotics might have the effect of the antioxidant ability, which is in line with previous studies [20, 22]. In this study, there was no significant difference in the level of TBIL before and after treatment in males in the AU subgroup and ANF subgroup, while female patients had lower TBIL after treatment, confirming that females were more susceptible to oxidative stress. Similar results were collected in previous studies [41, 42]. Although, it must be made clear that bilirubin is transported as an albumin binding complex in plasma, and the consumed plasma albumin may be the cause of the decrease in bilirubin.

There are a few limitations to this study. Firstly, the levels of peripheral albumin, bilirubin, and uric acid are susceptible to diet, but this study did not strictly control the dietary. A previous study did control the dietary factors but came up with similar findings [42]. Besides, the three non-enzymatic antioxidants can be affected by many factors, such as body mass index, particular exercise programs, glucose, and lipid metabolism [43, 44]. Even though some controls were made in this study, there are still some influences that cannot be excluded. Moreover, the amount of cigarette smoking can affect the oxidative status, but the study only collects the number of smokers, the specific number of cigarettes were not collected. Secondly, although previous studies showed that the antioxidant capacity of albumin, bilirubin, and uric acid accounted for more than 85% of the total antioxidant capacity of plasma, more indicators should be investigated to verify further the conclusion (such as total antioxidant capacity, lipid peroxides, ascorbic acid, and thiols). Thirdly, some studies suggested that the peripheral antioxidant capacity is consistent with the central nervous system [45]. However, the peripheral status is merely indirect evidence, which is not the same as the central nervous system. Fourthly, the study did not limit the treatment, so the types of antipsychotics in patients were various, and most of the patients were treated with two types of antipsychotics. Therefore, it is difficult for us to analyze the relationship between non-enzymatic antioxidant levels and a specific type of antipsychotic. Fifthly, 2/3 of the included patients were female in this study, which resulted in the selection bias. Future studies should improve this part. Finally, this study only performed a 12-week observation on the SCZ-AS patients, while a more extended observation is necessary. Beyond that, larger sample size can be expected for this study.

## Conclusions

This study observed that TBIL in SCZ was higher than that of HC and decreased after treatment, suggesting that patients with SCZ have a higher oxidative stress status both in the acute and remission stage. Antipsychotics may have an antioxidant effect. The increased level of UA in SCZ may be associated with the use of antipsychotics. Moreover, there may be a constant consumption of ALB during the acute and remission stage. In summary, the dysfunction of the peripheral non-enzymatic anti-oxidation system may be involved in the pathogenesis of SCZ, and females may be more susceptible to oxidative stress.

## Data Availability

The datasets used and/or analyzed during the current study are available from the corresponding author on reasonable request.

## Abbreviations

SCZ: schizophrenia
SCZ-AS: schizophrenic patients in the acute stage
SCZ-RS: schizophrenic patients in the remission stage
HC: healthy control
AU: antipsychotics-use
ANF: antipsychotics-naïve/free
UA: uric acid
ALB: albumin
TBIL: total bilirubin
PANSS: Positive and Negative Syndrome Scale
CGI-S: Clinical Global Impressions-Severity of Illness
DSM-5: Diagnostic and Statistical Manual of Mental Disorders, fifth edition
ROS: reactive oxygen species
RNS: reactive nitrogen species
MECT: modified electroconvulsive therapy
